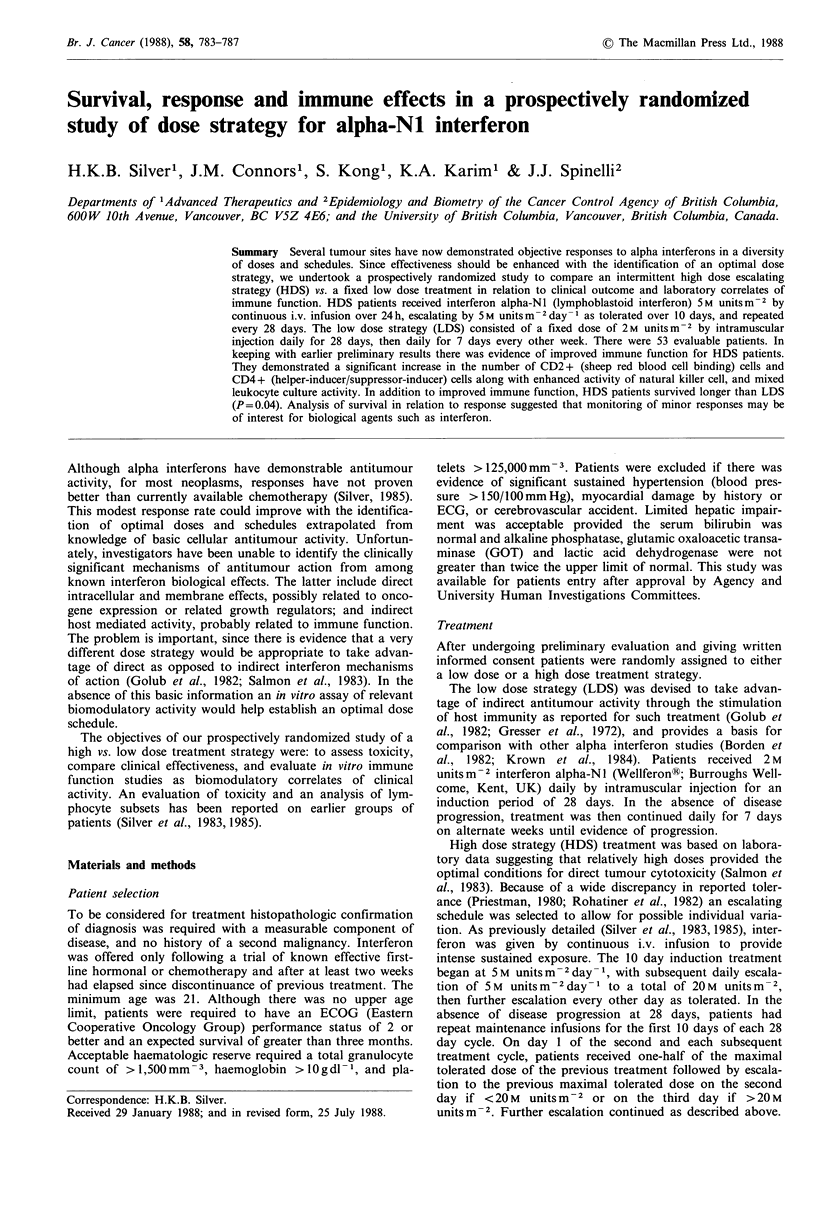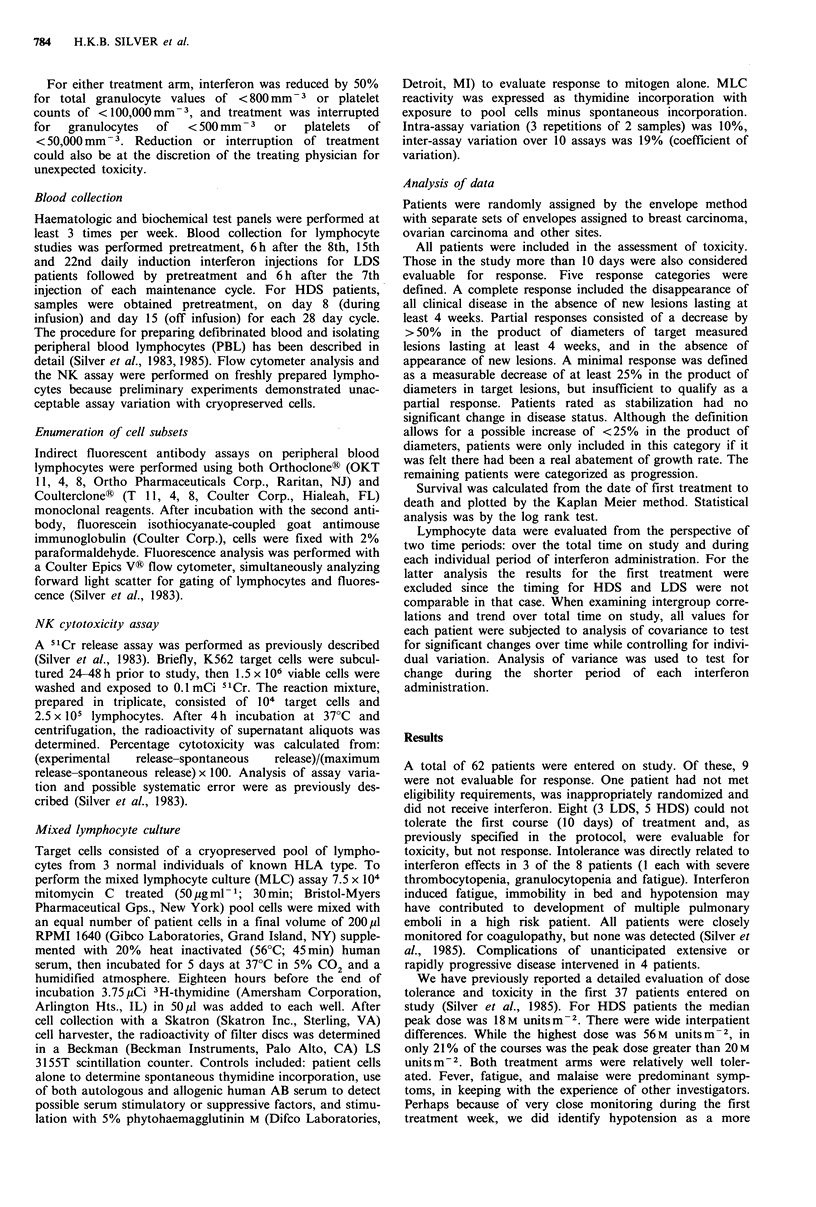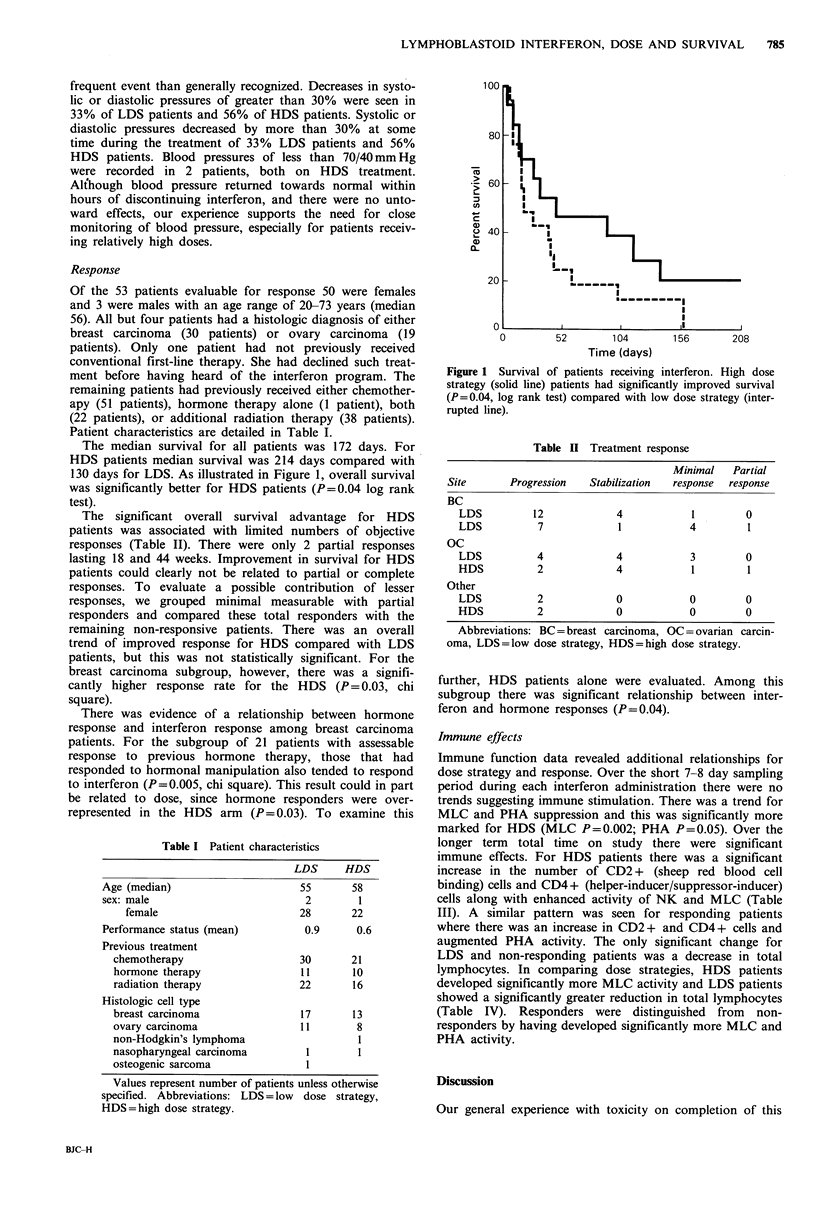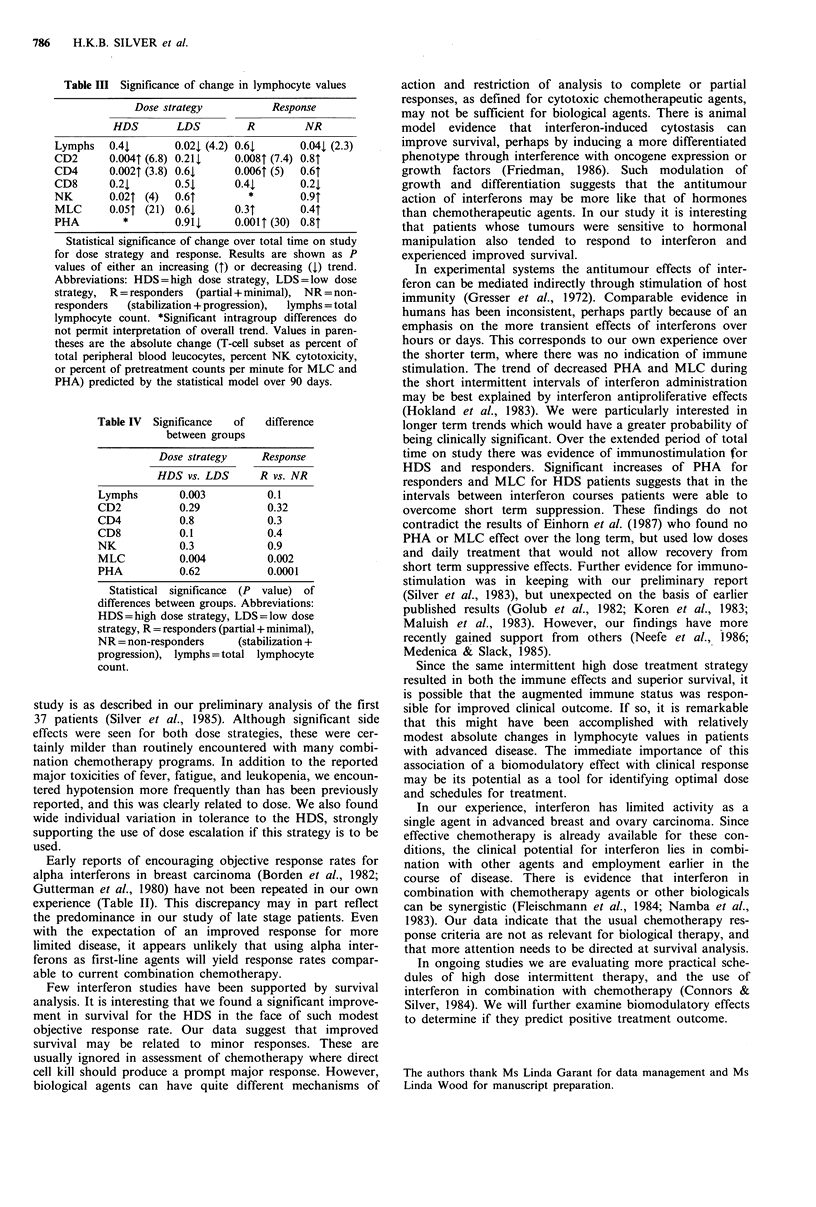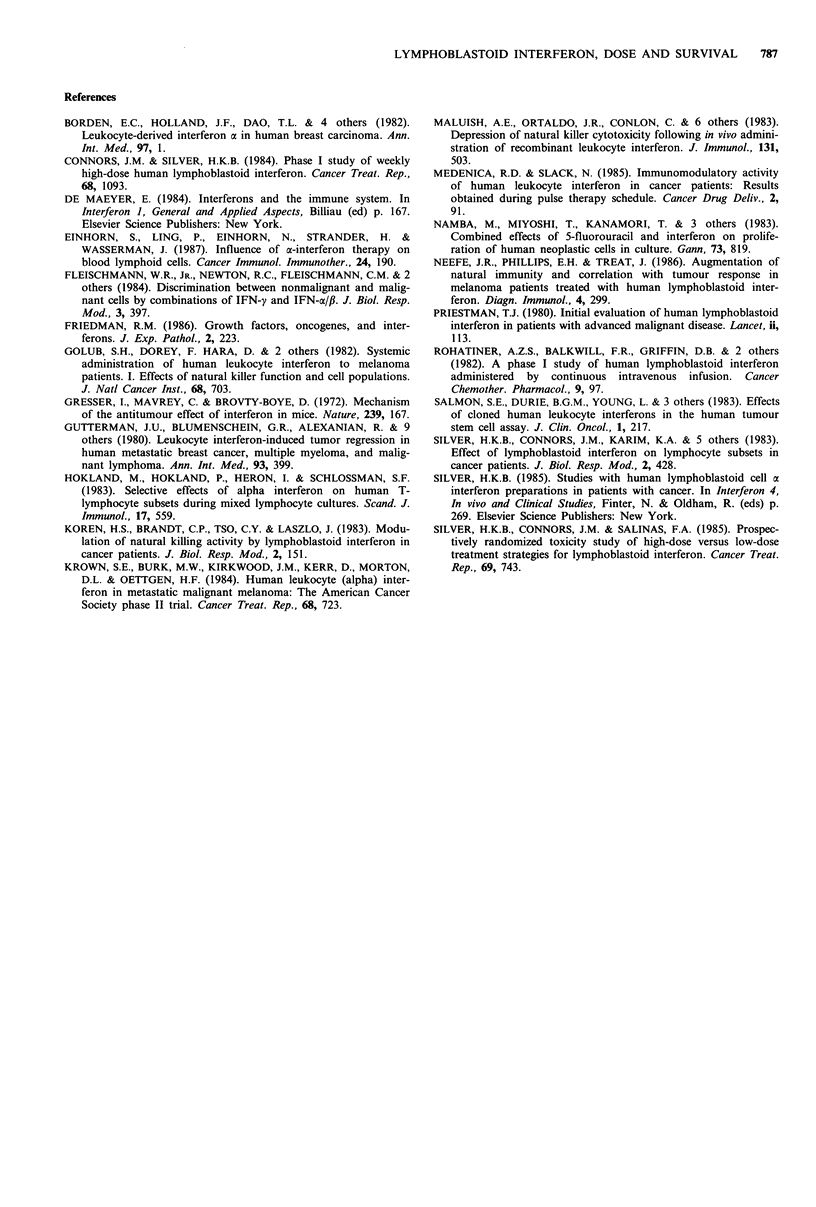# Survival, response and immune effects in a prospectively randomized study of dose strategy for alpha-N1 interferon.

**DOI:** 10.1038/bjc.1988.309

**Published:** 1988-12

**Authors:** H. K. Silver, J. M. Connors, S. Kong, K. A. Karim, J. J. Spinelli

**Affiliations:** Department of Advanced Therapeutics, University of British Columbia, Vancouver, Canada.

## Abstract

Several tumour sites have now demonstrated objective responses to alpha interferons in a diversity of doses and schedules. Since effectiveness should be enhanced with the identification of an optimal dose strategy, we undertook a prospectively randomized study to compare an intermittent high dose escalating strategy (HDS) vs. a fixed low dose treatment in relation to clinical outcome and laboratory correlates of immune function. HDS patients received interferon alpha-N1 (lymphoblastoid interferon) 5M units m-2 by continuous i.v. infusion over 24 h, escalating by 5 M units m-2 day-1 as tolerated over 10 days, and repeated every 28 days. The low dose strategy (LDS) consisted of a fixed dose of 2 M units m-2 by intramuscular injection daily for 28 days, then daily for 7 days every other week. There were 53 evaluable patients. In keeping with earlier preliminary results there was evidence of improved immune function for HDS patients. They demonstrated a significant increase in the number of CD2+ (sheep red blood cell binding) cells and CD4+ (helper-inducer/suppressor-inducer) cells along with enhanced activity of natural killer cell, and mixed leukocyte culture activity. In addition to improved immune function, HDS patients survived longer than LDS (P = 0.04). Analysis of survival in relation to response suggested that monitoring of minor responses may be of interest for biological agents such as interferon.


Br. J. Cancer (1988), 58, 783–787 © The Macmillan Press Ltd., 1988

### Survival, response and immune effects in a prospectively randomized study of dose strategy for alpha-N1 interferon

H.K.B. Silver[1], J.M. Connors[1], S. Kong[1], K.A. Karim[1] & J.J. Spinelli[2]

*Departments of [1]Advanced Therapeutics and [2]Epidemiology and Biometry of the Cancer Control Agency of British Columbia, 600W 10th Avenue, Vancouver, BC V5Z 4E6; and the University of British Columbia, Vancouver, British Columbia, Canada.*

**Summary** Several tumour sites have now demonstrated objective responses to alpha interferons in a diversity of doses and schedules. Since effectiveness should be enhanced with the identification of an optimal dose strategy, we undertook a prospectively randomized study to compare an intermittent high dose escalating strategy (HDS) *vs.* a fixed low dose treatment in relation to clinical outcome and laboratory correlates of immune function. HDS patients received interferon alpha-N1 (lymphoblastoid interferon) 5 M units $m^{-2}$ by continuous i.v. infusion over 24 h, escalating by 5 M units $m^{-2}$ $day^{-1}$ as tolerated over 10 days, and repeated every 28 days. The low dose strategy (LDS) consisted of a fixed dose of 2 M units $m^{-2}$ by intramuscular injection daily for 28 days, then daily for 7 days every other week. There were 53 evaluable patients. In keeping with earlier preliminary results there was evidence of improved immune function for HDS patients. They demonstrated a significant increase in the number of CD2+ (sheep red blood cell binding) cells and CD4+ (helper-inducer/suppressor-inducer) cells along with enhanced activity of natural killer cell, and mixed leukocyte culture activity. In addition to improved immune function, HDS patients survived longer than LDS ($P=0.04$). Analysis of survival in relation to response suggested that monitoring of minor responses may be of interest for biological agents such as interferon.

Although alpha interferons have demonstrable antitumour activity, for most neoplasms, responses have not proven better than currently available chemotherapy (Silver, 1985). This modest response rate could improve with the identification of optimal doses and schedules extrapolated from knowledge of basic cellular antitumour activity. Unfortunately, investigators have been unable to identify the clinically significant mechanisms of antitumour action from among known interferon biological effects. The latter include direct intracellular and membrane effects, possibly related to oncogene expression or related growth regulators; and indirect host mediated activity, probably related to immune function. The problem is important, since there is evidence that a very different dose strategy would be appropriate to take advantage of direct as opposed to indirect interferon mechanisms of action (Golub *et al.*, 1982; Salmon *et al.*, 1983). In the absence of this basic information an *in vitro* assay of relevant biomodulatory activity would help establish an optimal dose schedule.

The objectives of our prospectively randomized study of a high *vs.* low dose treatment strategy were: to assess toxicity, compare clinical effectiveness, and evaluate *in vitro* immune function studies as biomodulatory correlates of clinical activity. An evaluation of toxicity and an analysis of lymphocyte subsets has been reported on earlier groups of patients (Silver *et al.*, 1983, 1985).

### Materials and methods

### *Patient selection*

To be considered for treatment histopathologic confirmation of diagnosis was required with a measurable component of disease, and no history of a second malignancy. Interferon was offered only following a trial of known effective first-line hormonal or chemotherapy and after at least two weeks had elapsed since discontinuance of previous treatment. The minimum age was 21. Although there was no upper age limit, patients were required to have an ECOG (Eastern Cooperative Oncology Group) performance status of 2 or better and an expected survival of greater than three months. Acceptable haematologic reserve required a total granulocyte count of $>1{,}500\,mm^{-3}$, haemoglobin $>10\,g\,dl^{-1}$, and platelets $>125{,}000\,mm^{-3}$. Patients were excluded if there was evidence of significant sustained hypertension (blood pressure >150/100 mm Hg), myocardial damage by history or ECG, or cerebrovascular accident. Limited hepatic impairment was acceptable provided the serum bilirubin was normal and alkaline phosphatase, glutamic oxaloacetic transaminase (GOT) and lactic acid dehydrogenase were not greater than twice the upper limit of normal. This study was available for patients entry after approval by Agency and University Human Investigations Committees.

### *Treatment*

After undergoing preliminary evaluation and giving written informed consent patients were randomly assigned to either a low dose or a high dose treatment strategy.

The low dose strategy (LDS) was devised to take advantage of indirect antitumour activity through the stimulation of host immunity as reported for such treatment (Golub *et al.*, 1982; Gresser *et al.*, 1972), and provides a basis for comparison with other alpha interferon studies (Borden *et al.*, 1982; Krown *et al.*, 1984). Patients received 2 M units $m^{-2}$ interferon alpha-N1 (Wellferon®; Burroughs Wellcome, Kent, UK) daily by intramuscular injection for an induction period of 28 days. In the absence of disease progression, treatment was then continued daily for 7 days on alternate weeks until evidence of progression.

High dose strategy (HDS) treatment was based on laboratory data suggesting that relatively high doses provided the optimal conditions for direct tumour cytotoxicity (Salmon *et al.*, 1983). Because of a wide discrepancy in reported tolerance (Priestman, 1980; Rohatiner *et al.*, 1982) an escalating schedule was selected to allow for possible individual variation. As previously detailed (Silver *et al.*, 1983, 1985), interferon was given by continuous i.v. infusion to provide intense sustained exposure. The 10 day induction treatment began at 5 M units $m^{-2}$ $day^{-1}$, with subsequent daily escalation of 5 M units $m^{-2}$ $day^{-1}$ to a total of 20 M units $m^{-2}$, then further escalation every other day as tolerated. In the absence of disease progression at 28 days, patients had repeat maintenance infusions for the first 10 days of each 28 day cycle. On day 1 of the second and each subsequent treatment cycle, patients received one-half of the maximal tolerated dose of the previous treatment followed by escalation to the previous maximal tolerated dose on the second day if <20 M units $m^{-2}$ or on the third day if >20 M units $m^{-2}$. Further escalation continued as described above.

Correspondence: H.K.B. Silver.
Received 29 January 1988; and in revised form, 25 July 1988.

For either treatment arm, interferon was reduced by 50% for total granulocyte values of $<800\,mm^{-3}$ or platelet counts of $<100{,}000\,mm^{-3}$, and treatment was interrupted for granulocytes of $<500\,mm^{-3}$ or platelets of $<50{,}000\,mm^{-3}$. Reduction or interruption of treatment could also be at the discretion of the treating physician for unexpected toxicity.

*Blood collection*

Haematologic and biochemical test panels were performed at least 3 times per week. Blood collection for lymphocyte studies was performed pretreatment, 6 h after the 8th, 15th and 22nd daily induction interferon injections for LDS patients followed by pretreatment and 6 h after the 7th injection of each maintenance cycle. For HDS patients, samples were obtained pretreatment, on day 8 (during infusion) and day 15 (off infusion) for each 28 day cycle. The procedure for preparing defibrinated blood and isolating peripheral blood lymphocytes (PBL) has been described in detail (Silver *et al.*, 1983, 1985). Flow cytometer analysis and the NK assay were performed on freshly prepared lymphocytes because preliminary experiments demonstrated unacceptable assay variation with cryopreserved cells.

*Enumeration of cell subsets*

Indirect fluorescent antibody assays on peripheral blood lymphocytes were performed using both Orthoclone® (OKT 11, 4, 8, Ortho Pharmaceuticals Corp., Raritan, NJ) and Coulterclone® (T 11, 4, 8, Coulter Corp., Hialeah, FL) monoclonal reagents. After incubation with the second antibody, fluorescein isothiocyanate-coupled goat antimouse immunoglobulin (Coulter Corp.), cells were fixed with 2% paraformaldehyde. Fluorescence analysis was performed with a Coulter Epics V® flow cytometer, simultaneously analyzing forward light scatter for gating of lymphocytes and fluorescence (Silver *et al.*, 1983).

*NK cytotoxicity assay*

A $^{51}Cr$ release assay was performed as previously described (Silver *et al.*, 1983). Briefly, K562 target cells were subcultured 24–48 h prior to study, then $1.5\times10^{6}$ viable cells were washed and exposed to 0.1 mCi $^{51}Cr$. The reaction mixture, prepared in triplicate, consisted of $10^{4}$ target cells and $2.5\times10^{5}$ lymphocytes. After 4 h incubation at 37°C and centrifugation, the radioactivity of supernatant aliquots was determined. Percentage cytotoxicity was calculated from: (experimental release–spontaneous release)/(maximum release–spontaneous release) × 100. Analysis of assay variation and possible systematic error were as previously described (Silver *et al.*, 1983).

*Mixed lymphocyte culture*

Target cells consisted of a cryopreserved pool of lymphocytes from 3 normal individuals of known HLA type. To perform the mixed lymphocyte culture (MLC) assay $7.5\times10^{4}$ mitomycin C treated ($50\,\mu g\,ml^{-1}$; 30 min; Bristol-Myers Pharmaceutical Gps., New York) pool cells were mixed with an equal number of patient cells in a final volume of 200 µl RPMI 1640 (Gibco Laboratories, Grand Island, NY) supplemented with 20% heat inactivated (56°C; 45 min) human serum, then incubated for 5 days at 37°C in 5% $CO_2$ and a humidified atmosphere. Eighteen hours before the end of incubation 3.75 µCi $^{3}H$-thymidine (Amersham Corporation, Arlington Hts., IL) in 50 µl was added to each well. After cell collection with a Skatron (Skatron Inc., Sterling, VA) cell harvester, the radioactivity of filter discs was determined in a Beckman (Beckman Instruments, Palo Alto, CA) LS 3155T scintillation counter. Controls included: patient cells alone to determine spontaneous thymidine incorporation, use of both autologous and allogenic human AB serum to detect possible serum stimulatory or suppressive factors, and stimulation with 5% phytohaemagglutinin M (Difco Laboratories, Detroit, MI) to evaluate response to mitogen alone. MLC reactivity was expressed as thymidine incorporation with exposure to pool cells minus spontaneous incorporation. Intra-assay variation (3 repetitions of 2 samples) was 10%, inter-assay variation over 10 assays was 19% (coefficient of variation).

*Analysis of data*

Patients were randomly assigned by the envelope method with separate sets of envelopes assigned to breast carcinoma, ovarian carcinoma and other sites.

All patients were included in the assessment of toxicity. Those in the study more than 10 days were also considered evaluable for response. Five response categories were defined. A complete response included the disappearance of all clinical disease in the absence of new lesions lasting at least 4 weeks. Partial responses consisted of a decrease by >50% in the product of diameters of target measured lesions lasting at least 4 weeks, and in the absence of appearance of new lesions. A minimal response was defined as a measurable decrease of at least 25% in the product of diameters in target lesions, but insufficient to qualify as a partial response. Patients rated as stabilization had no significant change in disease status. Although the definition allows for a possible increase of <25% in the product of diameters, patients were only included in this category if it was felt there had been a real abatement of growth rate. The remaining patients were categorized as progression.

Survival was calculated from the date of first treatment to death and plotted by the Kaplan Meier method. Statistical analysis was by the log rank test.

Lymphocyte data were evaluated from the perspective of two time periods: over the total time on study and during each individual period of interferon administration. For the latter analysis the results for the first treatment were excluded since the timing for HDS and LDS were not comparable in that case. When examining intergroup correlations and trend over total time on study, all values for each patient were subjected to analysis of covariance to test for significant changes over time while controlling for individual variation. Analysis of variance was used to test for change during the shorter period of each interferon administration.

### Results

A total of 62 patients were entered on study. Of these, 9 were not evaluable for response. One patient had not met eligibility requirements, was inappropriately randomized and did not receive interferon. Eight (3 LDS, 5 HDS) could not tolerate the first course (10 days) of treatment and, as previously specified in the protocol, were evaluable for toxicity, but not response. Intolerance was directly related to interferon effects in 3 of the 8 patients (1 each with severe thrombocytopenia, granulocytopenia and fatigue). Interferon induced fatigue, immobility in bed and hypotension may have contributed to development of multiple pulmonary emboli in a high risk patient. All patients were closely monitored for coagulopathy, but none was detected (Silver *et al.*, 1985). Complications of unanticipated extensive or rapidly progressive disease intervened in 4 patients.

We have previously reported a detailed evaluation of dose tolerance and toxicity in the first 37 patients entered on study (Silver *et al.*, 1985). For HDS patients the median peak dose was $18\,\mathrm{M}$ units $m^{-2}$. There were wide interpatient differences. While the highest dose was $56\,\mathrm{M}$ units $m^{-2}$, in only 21% of the courses was the peak dose greater than $20\,\mathrm{M}$ units $m^{-2}$. Both treatment arms were relatively well tolerated. Fever, fatigue, and malaise were predominant symptoms, in keeping with the experience of other investigators. Perhaps because of very close monitoring during the first treatment week, we did identify hypotension as a more frequent event than generally recognized. Decreases in systolic or diastolic pressures of greater than 30% were seen in 33% of LDS patients and 56% of HDS patients. Systolic or diastolic pressures decreased by more than 30% at some time during the treatment of 33% LDS patients and 56% HDS patients. Blood pressures of less than 70/40 mm Hg were recorded in 2 patients, both on HDS treatment. Although blood pressure returned towards normal within hours of discontinuing interferon, and there were no untoward effects, our experience supports the need for close monitoring of blood pressure, especially for patients receiving relatively high doses.

*Response*

Of the 53 patients evaluable for response 50 were females and 3 were males with an age range of 20–73 years (median 56). All but four patients had a histologic diagnosis of either breast carcinoma (30 patients) or ovary carcinoma (19 patients). Only one patient had not previously received conventional first-line therapy. She had declined such treatment before having heard of the interferon program. The remaining patients had previously received either chemotherapy (51 patients), hormone therapy alone (1 patient), both (22 patients), or additional radiation therapy (38 patients). Patient characteristics are detailed in Table I.

The median survival for all patients was 172 days. For HDS patients median survival was 214 days compared with 130 days for LDS. As illustrated in Figure 1, overall survival was significantly better for HDS patients ($P = 0.04$ log rank test).

The significant overall survival advantage for HDS patients was associated with limited numbers of objective responses (Table II). There were only 2 partial responses lasting 18 and 44 weeks. Improvement in survival for HDS patients could clearly not be related to partial or complete responses. To evaluate a possible contribution of lesser responses, we grouped minimal measurable with partial responders and compared these total responders with the remaining non-responsive patients. There was an overall trend of improved response for HDS compared with LDS patients, but this was not statistically significant. For the breast carcinoma subgroup, however, there was a significantly higher response rate for the HDS ($P = 0.03$, chi square).

There was evidence of a relationship between hormone response and interferon response among breast carcinoma patients. For the subgroup of 21 patients with assessable response to previous hormone therapy, those that had responded to hormonal manipulation also tended to respond to interferon ($P = 0.005$, chi square). This result could in part be related to dose, since hormone responders were over-represented in the HDS arm ($P = 0.03$). To examine this further, HDS patients alone were evaluated. Among this subgroup there was significant relationship between interferon and hormone responses ($P = 0.04$).

**Table I** Patient characteristics

| | *LDS* | *HDS* |
|---|---|---|
| Age (median) | 55 | 58 |
| sex: male | 2 | 1 |
| female | 28 | 22 |
| Performance status (mean) | 0.9 | 0.6 |
| Previous treatment | | |
| chemotherapy | 30 | 21 |
| hormone therapy | 11 | 10 |
| radiation therapy | 22 | 16 |
| Histologic cell type | | |
| breast carcinoma | 17 | 13 |
| ovary carcinoma | 11 | 8 |
| non-Hodgkin's lymphoma | | 1 |
| nasopharyngeal carcinoma | 1 | 1 |
| osteogenic sarcoma | 1 | |

Values represent number of patients unless otherwise specified. Abbreviations: LDS = low dose strategy, HDS = high dose strategy.

**Figure 1** Survival of patients receiving interferon. High dose strategy (solid line) patients had significantly improved survival ($P = 0.04$, log rank test) compared with low dose strategy (interrupted line).

**Table II** Treatment response

| *Site* | *Progression* | *Stabilization* | *Minimal response* | *Partial response* |
|---|---|---|---|---|
| BC | | | | |
| LDS | 12 | 4 | 1 | 0 |
| LDS | 7 | 1 | 4 | 1 |
| OC | | | | |
| LDS | 4 | 4 | 3 | 0 |
| HDS | 2 | 4 | 1 | 1 |
| Other | | | | |
| LDS | 2 | 0 | 0 | 0 |
| HDS | 2 | 0 | 0 | 0 |

Abbreviations: BC = breast carcinoma, OC = ovarian carcinoma, LDS = low dose strategy, HDS = high dose strategy.

*Immune effects*

Immune function data revealed additional relationships for dose strategy and response. Over the short 7–8 day sampling period during each interferon administration there were no trends suggesting immune stimulation. There was a trend for MLC and PHA suppression and this was significantly more marked for HDS (MLC $P = 0.002$; PHA $P = 0.05$). Over the longer term total time on study there were significant immune effects. For HDS patients there was a significant increase in the number of CD2+ (sheep red blood cell binding) cells and CD4+ (helper-inducer/suppressor-inducer) cells along with enhanced activity of NK and MLC (Table III). A similar pattern was seen for responding patients where there was an increase in CD2+ and CD4+ cells and augmented PHA activity. The only significant change for LDS and non-responding patients was a decrease in total lymphocytes. In comparing dose strategies, HDS patients developed significantly more MLC activity and LDS patients showed a significantly greater reduction in total lymphocytes (Table IV). Responders were distinguished from non-responders by having developed significantly more MLC and PHA activity.

### Discussion

Our general experience with toxicity on completion of this

**Table III** Significance of change in lymphocyte values

| | Dose strategy | | Response | |
|---|---|---|---|---|
| | *HDS* | *LDS* | *R* | *NR* |
| Lymphs | 0.4↓ | 0.02↓ (4.2) | 0.6↓ | 0.04↓ (2.3) |
| CD2 | 0.004↑ (6.8) | 0.21↓ | 0.008↑ (7.4) | 0.8↑ |
| CD4 | 0.002↑ (3.8) | 0.6↓ | 0.006↑ (5) | 0.6↑ |
| CD8 | 0.2↓ | 0.5↓ | 0.4↓ | 0.2↓ |
| NK | 0.02↑ (4) | 0.6↑ | * | 0.9↑ |
| MLC | 0.05↑ (21) | 0.6↓ | 0.3↑ | 0.4↑ |
| PHA | * | 0.91↓ | 0.001↑ (30) | 0.8↑ |

Statistical significance of change over total time on study for dose strategy and response. Results are shown as $P$ values of either an increasing (↑) or decreasing (↓) trend. Abbreviations: HDS = high dose strategy, LDS = low dose strategy, R = responders (partial + minimal), NR = non-responders (stabilization + progression), lymphs = total lymphocyte count. *Significant intragroup differences do not permit interpretation of overall trend. Values in parentheses are the absolute change (T-cell subset as percent of total peripheral blood leucocytes, percent NK cytotoxicity, or percent of pretreatment counts per minute for MLC and PHA) predicted by the statistical model over 90 days.

**Table IV** Significance of difference between groups

| | Dose strategy | Response |
|---|---|---|
| | *HDS vs. LDS* | *R vs. NR* |
| Lymphs | 0.003 | 0.1 |
| CD2 | 0.29 | 0.32 |
| CD4 | 0.8 | 0.3 |
| CD8 | 0.1 | 0.4 |
| NK | 0.3 | 0.9 |
| MLC | 0.004 | 0.002 |
| PHA | 0.62 | 0.0001 |

Statistical significance ($P$ value) of differences between groups. Abbreviations: HDS = high dose strategy, LDS = low dose strategy, R = responders (partial + minimal), NR = non-responders (stabilization + progression), lymphs = total lymphocyte count.

study is as described in our preliminary analysis of the first 37 patients (Silver *et al.*, 1985). Although significant side effects were seen for both dose strategies, these were certainly milder than routinely encountered with many combination chemotherapy programs. In addition to the reported major toxicities of fever, fatigue, and leukopenia, we encountered hypotension more frequently than has been previously reported, and this was clearly related to dose. We also found wide individual variation in tolerance to the HDS, strongly supporting the use of dose escalation if this strategy is to be used.

Early reports of encouraging objective response rates for alpha interferons in breast carcinoma (Borden *et al.*, 1982; Gutterman *et al.*, 1980) have not been repeated in our own experience (Table II). This discrepancy may in part reflect the predominance in our study of late stage patients. Even with the expectation of an improved response for more limited disease, it appears unlikely that using alpha interferons as first-line agents will yield response rates comparable to current combination chemotherapy.

Few interferon studies have been supported by survival analysis. It is interesting that we found a significant improvement in survival for the HDS in the face of such modest objective response rate. Our data suggest that improved survival may be related to minor responses. These are usually ignored in assessment of chemotherapy where direct cell kill should produce a prompt major response. However, biological agents can have quite different mechanisms of action and restriction of analysis to complete or partial responses, as defined for cytotoxic chemotherapeutic agents, may not be sufficient for biological agents. There is animal model evidence that interferon-induced cytostasis can improve survival, perhaps by inducing a more differentiated phenotype through interference with oncogene expression or growth factors (Friedman, 1986). Such modulation of growth and differentiation suggests that the antitumour action of interferons may be more like that of hormones than chemotherapeutic agents. In our study it is interesting that patients whose tumours were sensitive to hormonal manipulation also tended to respond to interferon and experienced improved survival.

In experimental systems the antitumour effects of interferon can be mediated indirectly through stimulation of host immunity (Gresser *et al.*, 1972). Comparable evidence in humans has been inconsistent, perhaps partly because of an emphasis on the more transient effects of interferons over hours or days. This corresponds to our own experience over the shorter term, where there was no indication of immune stimulation. The trend of decreased PHA and MLC during the short intermittent intervals of interferon administration may be best explained by interferon antiproliferative effects (Hokland *et al.*, 1983). We were particularly interested in longer term trends which would have a greater probability of being clinically significant. Over the extended period of total time on study there was evidence of immunostimulation for HDS and responders. Significant increases of PHA for responders and MLC for HDS patients suggests that in the intervals between interferon courses patients were able to overcome short term suppression. These findings do not contradict the results of Einhorn *et al.* (1987) who found no PHA or MLC effect over the long term, but used low doses and daily treatment that would not allow recovery from short term suppressive effects. Further evidence for immunostimulation was in keeping with our preliminary report (Silver *et al.*, 1983), but unexpected on the basis of earlier published results (Golub *et al.*, 1982; Koren *et al.*, 1983; Maluish *et al.*, 1983). However, our findings have more recently gained support from others (Neefe *et al.*, 1986; Medenica & Slack, 1985).

Since the same intermittent high dose treatment strategy resulted in both the immune effects and superior survival, it is possible that the augmented immune status was responsible for improved clinical outcome. If so, it is remarkable that this might have been accomplished with relatively modest absolute changes in lymphocyte values in patients with advanced disease. The immediate importance of this association of a biomodulatory effect with clinical response may be its potential as a tool for identifying optimal dose and schedules for treatment.

In our experience, interferon has limited activity as a single agent in advanced breast and ovary carcinoma. Since effective chemotherapy is already available for these conditions, the clinical potential for interferon lies in combination with other agents and employment earlier in the course of disease. There is evidence that interferon in combination with chemotherapy agents or other biologicals can be synergistic (Fleischmann *et al.*, 1984; Namba *et al.*, 1983). Our data indicate that the usual chemotherapy response criteria are not as relevant for biological therapy, and that more attention needs to be directed at survival analysis.

In ongoing studies we are evaluating more practical schedules of high dose intermittent therapy, and the use of interferon in combination with chemotherapy (Connors & Silver, 1984). We will further examine biomodulatory effects to determine if they predict positive treatment outcome.

The authors thank Ms Linda Garant for data management and Ms Linda Wood for manuscript preparation.

### References

BORDEN, E.C., HOLLAND, J.F., DAO, T.L. & 4 others (1982). Leukocyte-derived interferon α in human breast carcinoma. *Ann. Int. Med.*, **97**, 1.

CONNORS, J.M. & SILVER, H.K.B. (1984). Phase I study of weekly high-dose human lymphoblastoid interferon. *Cancer Treat. Rep.*, **68**, 1093.

DE MAEYER, E. (1984). Interferons and the immune system. In *Interferon 1, General and Applied Aspects*, Billiau (ed) p. 167. Elsevier Science Publishers: New York.

EINHORN, S., LING, P., EINHORN, N., STRANDER, H. & WASSERMAN, J. (1987). Influence of α-interferon therapy on blood lymphoid cells. *Cancer Immunol. Immunother.*, **24**, 190.

FLEISCHMANN, W.R., JR., NEWTON, R.C., FLEISCHMANN, C.M. & 2 others (1984). Discrimination between nonmalignant and malignant cells by combinations of IFN-γ and IFN-α/β. *J. Biol. Resp. Mod.*, **3**, 397.

FRIEDMAN, R.M. (1986). Growth factors, oncogenes, and interferons. *J. Exp. Pathol.*, **2**, 223.

GOLUB, S.H., DOREY, F. HARA, D. & 2 others (1982). Systemic administration of human leukocyte interferon to melanoma patients. I. Effects of natural killer function and cell populations. *J. Natl Cancer Inst.*, **68**, 703.

GRESSER, I., MAVREY, C. & BROVTY-BOYE, D. (1972). Mechanism of the antitumour effect of interferon in mice. *Nature*, **239**, 167.

GUTTERMAN, J.U., BLUMENSCHEIN, G.R., ALEXANIAN, R. & 9 others (1980). Leukocyte interferon-induced tumor regression in human metastatic breast cancer, multiple myeloma, and malignant lymphoma. *Ann. Int. Med.*, **93**, 399.

HOKLAND, M., HOKLAND, P., HERON, I. & SCHLOSSMAN, S.F. (1983). Selective effects of alpha interferon on human T-lymphocyte subsets during mixed lymphocyte cultures. *Scand. J. Immunol.*, **17**, 559.

KOREN, H.S., BRANDT, C.P., TSO, C.Y. & LASZLO, J. (1983). Modulation of natural killing activity by lymphoblastoid interferon in cancer patients. *J. Biol. Resp. Mod.*, **2**, 151.

KROWN, S.E., BURK, M.W., KIRKWOOD, J.M., KERR, D., MORTON, D.L. & OETTGEN, H.F. (1984). Human leukocyte (alpha) interferon in metastatic malignant melanoma: The American Cancer Society phase II trial. *Cancer Treat. Rep.*, **68**, 723.

MALUISH, A.E., ORTALDO, J.R., CONLON, C. & 6 others (1983). Depression of natural killer cytotoxicity following *in vivo* administration of recombinant leukocyte interferon. *J. Immunol.*, **131**, 503.

MEDENICA, R.D. & SLACK, N. (1985). Immunomodulatory activity of human leukocyte interferon in cancer patients: Results obtained during pulse therapy schedule. *Cancer Drug Deliv.*, **2**, 91.

NAMBA, M., MIYOSHI, T., KANAMORI, T. & 3 others (1983). Combined effects of 5-fluorouracil and interferon on proliferation of human neoplastic cells in culture. *Gann*, **73**, 819.

NEEFE, J.R., PHILLIPS, E.H. & TREAT, J. (1986). Augmentation of natural immunity and correlation with tumour response in melanoma patients treated with human lymphoblastoid interferon. *Diagn. Immunol.*, **4**, 299.

PRIESTMAN, T.J. (1980). Initial evaluation of human lymphoblastoid interferon in patients with advanced malignant disease. *Lancet*, **ii**, 113.

ROHATINER, A.Z.S., BALKWILL, F.R., GRIFFIN, D.B. & 2 others (1982). A phase I study of human lymphoblastoid interferon administered by continuous intravenous infusion. *Cancer Chemother. Pharmacol.*, **9**, 97.

SALMON, S.E., DURIE, B.G.M., YOUNG, L. & 3 others (1983). Effects of cloned human leukocyte interferons in the human tumour stem cell assay. *J. Clin. Oncol.*, **1**, 217.

SILVER, H.K.B., CONNORS, J.M., KARIM, K.A. & 5 others (1983). Effect of lymphoblastoid interferon on lymphocyte subsets in cancer patients. *J. Biol. Resp. Mod.*, **2**, 428.

SILVER, H.K.B. (1985). Studies with human lymphoblastoid cell α interferon preparations in patients with cancer. In *Interferon 4, In vivo and Clinical Studies*, Finter, N. & Oldham, R. (eds) p. 269. Elsevier Science Publishers: New York.

SILVER, H.K.B., CONNORS, J.M. & SALINAS, F.A. (1985). Prospectively randomized toxicity study of high-dose versus low-dose treatment strategies for lymphoblastoid interferon. *Cancer Treat. Rep.*, **69**, 743.